# Enhanced Bioactivity of Rosemary, Sage, Lavender, and Chamomile Essential Oils by Fractionation, Combination, and Emulsification

**DOI:** 10.1021/acsomega.2c07508

**Published:** 2023-03-14

**Authors:** Nursenem Karaca, Betül Demirci, Mohsen Gavahian, Fatih Demirci

**Affiliations:** † Department of Pharmacognosy, Graduate School of Health Sciences, Anadolu University, Eskisehir 26470, Türkiye; ‡ Department of Pharmacognosy, Faculty of Pharmacy, Anadolu University, Eskisehir 26470, Türkiye; § Department of Food Science, National Pingtung University of Science and Technology, 1, Shuefu Road, Neipu, Pingtung 91201, Taiwan, ROC; ∥ Faculty of Pharmacy, Eastern Mediterranean University, N.Cyprus, Mersin 10, Famagusta 99628, Türkiye

## Abstract

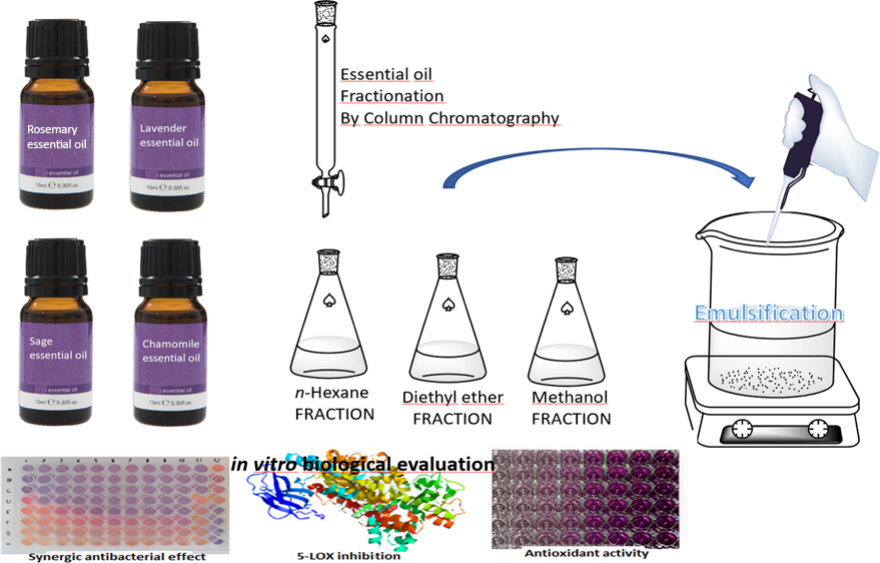

This study aimed to increase the bioactivity of essential oils by fractionation, combination, and emulsification. In this regard, pharmaceutical quality *Rosmarinus officinalis* L. (rosemary), *Salvia sclarea* L. (clary sage), *Lavandula latifolia* Medik. (spike lavender), and *Matricaria chamomilla* L. (chamomile) essential oils were fractionated by vacuum-column chromatography. The main components of the essential oils were verified, and their fractions were characterized by thin layer chromatography, gas chromatography-flame ionization detector, and gas chromatography/mass spectrometry. Besides, oil-in-water (O/W) emulsions of essential oils and diethyl ether fractions were obtained by the self-emulsification method, followed by droplet size, polydispersity index, and zeta potential value measurements. The in vitro antibacterial effects of the emulsions and binary combinations (10:90, 20:80, 30:70, 40:60, 50:50, 60:40, 70:30, 80:20, 90:10, v:v) against *Staphylococcus aureus* were determined by microdilution. In addition, the in vitro anti-biofilm, antioxidant, and anti-inflammatory effects of emulsion formulations were evaluated. According to the experimental results, fractionation and emulsification enhanced essential oil in vitro antibacterial, anti-inflammatory, and antioxidant effects due to increased solubility and nano-sized droplets. Among 22 different emulsion combinations, 1584 test concentrations resulted in 21 cases of synergistic effects. The mechanism of the increase in biological activities was hypothesized to be higher solubility and stability of the essential oil fractions. Food and pharmaceutical industries may benefit from the procedure proposed in this study.

## 1 Introduction

Emulsions are systems in which one liquid is dispersed in another liquid/s and contains droplets with an average radius of 100 nm–100 mm.^1−3^ As is well known, there are two commonly used emulsions: oil-in-water (O/W) and water-in-oil (W/O) emulsions. Considering the solubility, W/O and O/W nanoemulsions are better for hydrophilic and lipophilic drugs, respectively. In recent years, O/W nanoemulsions are used in health, cosmetics, food, agricultural chemistry, pharmaceuticals, and biotechnology, among other sectors.^4,5^ It is known that stable nanoemulsions with a small droplet size can be produced by spontaneous emulsification by mixing and gradually adding water phase to the oil/surfactant mixture at constant temperature.^2,6,7^

As has been known for centuries, herbal materials and essential oils are used for the prevention and treatment of various diseases. Also, their products, such as essential oils, have broad biological activities such as antimicrobial, antioxidant, anti-inflammatory, analgesic, mucolytic, and stimulant. They have applications in perfumes, cosmetic preparations, insecticides, food preservatives, pharmaceuticals, and aromatherapy.^8,9^ However, their application in medicines is limited due to their low water solubility and instability, high volatility, and side effects. According to the literature, emulsification of lipophilic active substances can increase their stability, solubility, and biological effects.^10−13^ Although several essential oils have been fractionated,^9,14^ information on the fractionation for enhancing biological activity is a novel research area.

*Rosmarinus officinalis* L. (rosemary, Lamiaceae) rosemary contains antioxidant, antimicrobial, and anti-inflammatory bioactive substances. The main components of the essential oil obtained by hydrodistillation are 1,8-cineole, camphor, and α-pinene. Rosemary oil can treat acne, baldness, rheumatic pain, and circulatory obstruction. It is used for its carminative, diuretic, expectorant, and antispasmodic effects. In addition, rosemary oil has a pronounced impact on the central nervous system associated with brain stimulants and memory development.^15,16^

*Lavandula* essential oils are used in both cosmetics and therapy for centuries. The common species with antiseptic properties are *L. angustifolia,L. latifolia,L. stoechas,* and *L. x intermedia*. Lavender oil is often used as a relaxant, carminative, and sedative in aromatherapy. Lavender essential oils are known to be antibacterial, antifungal, carminative, sedative, and antidepressant for burns and effective against insect bites. More than 300 components were identified in *Lavandula latifolia* Medik. (spike lavender) essential oil, where the main components include linalool, 1,8-cineole, and camphor.^17,18^

*Salvia sclarea* L. (clary sage, Lamiaceae) is one of the most prevalent *Salvia*. Herbal part preparations of this plant are used as antidiarrheal and sedatives in Turkish folk medicine. Flower and leaf oil are used as a sedative to treat stomach pain, constipation, and sweating.^19^ The plant is also cultivated worldwide, especially in the Mediterranean and Central Europe. Essential oils obtained from *S. sclarea* are used as antidepressants, antiseptics, antispasmodic, carminative, and aphrodisiacs. Antifungal,^20,21^ anti-inflammatory and analgesic,^22^ antibacterials,^23^ and antioxidant effects were among the reported.

*Matricaria chamomilla* L. (chamomile, Asteraceae) is a popular medicinal plant. The chamomile preparations are known and used as herbal medicine for thousands of years. Chamomile tea prepared with dried flowers of *M. chamomilla* is widely consumed.^24^ Essential oil has traditionally been used as a cholagogue, carminative, anti-inflammatory, analgesic, and diuretic. Chamomile has been used as herbal medicine since ancient times and as anti-inflammatory and antiseptic agents, as well as antispasmodic, among other applications.^24,25^

This present study aims to enhance selected essential oil bioactivity by fractionation, combination, and emulsification. Also, it aims to determine the in vitro antimicrobial, anti-biofilm, anti-inflammatory, and antioxidant properties of essential oil-based products compared with freshly extracted oils. To the best of our knowledge, this study is the first report on using pharmacopeia-grade essential oils and their fractions, emulsions, and combinations from rosemary, sage, lavender, and chamomile for enhancing bioactivity.

## 2 Results and Discussion

### 2.1 Fractionation Yields and Chromatographic Analysis

Detailed gas chromatography (GC) and gas chromatography/mass spectrometry (GC/MS) analyses confirmed the pharma quality of the study material essential oils. The amounts and percentage yields were obtained by fractionating the essential oils with *n*-hexane, diethyl ether, and methanol (Table 1). To the best of our knowledge, this is the first report on the fractionation of essential oils before emulsification to enhance solubility and activity.

**Table 1:** Fraction Quantities and % Yields Obtained from Essential Oils^a^

essential oils	solvent of fractionation	fraction (g)	% yield
*Lavandula latifolia*	*n*-hexane	0.5	100
	diethyl ether	5.5	
	methanol	0	
*Rosmarinus officinalis*	*n*-hexane	1.2	91.2
	diethyl ether	4.3	
	methanol	-	
*Salvia sclarea*	*n*-hexane	0.1	97.5
	diethyl ether	5.6	
	methanol	0.1	
*Matricaria chamomilla*	*n*-hexane	0.9	100
	diethyl ether	5.1	
	methanol	-	

a g, gram; -, ≤0.001 g.

The chemical composition of the essential oil fractions separated was determined by thin layer chromatography (TLC), gas chromatography-flame ionization detector (GC-FID), and GC/MS methods according to their polarity. TLC evaluations were compared with standards, and results are listed in the Supporting Information. The main components of essential oils and fractions were determined by the GC-FID and GC/MS results.

The *L. latifolia* essential oil, *n*-hexane, and diethyl ether fraction compositions are listed comparatively in Table 2.

**Table 2:** Chemical Composition of *L. latifolia* Essential Oil, *n*-Hexane, and Diethyl Ether Fractions^a^

		%		
RRI	compound	*L. latifolia* essential oil	*n*-hexane fraction	diethyl ether fraction
1014	tricyclene	**T**	0.1	
1032	α-pinene	2.4	**20.4**	
1076	camphene	0.5	4.0	
1118	β-pinene	2.0	**19.8**	
1132	sabinene	T	0.3	
1159	δ-3-carene		0.1	
1174	myrcene	0.4	3.8	
1188	α*-*terpinene		0.1	
1203	limonene	1.3	**16.0**	
1213	1,8-cineole	**25.6**		**27.0**
1218	β-phellandrene		0.3	
1246	(*Z*)-β-ocimene	0.1	1.1	
1255	γ-terpinene	T	0.4	
1266	(*E*)-β-ocimene	0.1	1.3	
1280	*p*-cymene	0.6	7.0	
1290	terpinolene	t	0.4	
1439	γ-campholene aldehyde	t		0.3
1450	*trans-*linalool oxide (*furanoid*)	t		0.1
1478	*cis*-linalool oxide (*furanoid*)	t		t
1497	α-copaene		0.2	
1532	camphor	12.2		14.0
1535	β-bourbonene		0.6	
1549	β-cubebene		0.1	
1553	linalool	**45.2**		**49.0**
1565	linalyl acetate	2.3		1.8
1568	*trans*-α-bergamotene		0.1	
1572	α-bergamotene		0.2	
1611	terpinen-4-ol	0.2		0.2
1612	β-caryophyllene	1.0	15.9	
1617	lavandulyl acetate	0.1		
1668	*(Z)*-β-farnesene		0.8	
1669	sesquisabinene		0.1	
1684	isoborneol	0.5		0.6
1687	α-humulene	t	1.7	
1706	α-terpineol	1.5		1.6
1715	γ*-*terpineol			0.1
1719	borneol	0.9		1.0
1726	germacrene D	t	0.4	
1733	neryl acetate	0.3		0.4
1741	β-bisabolene		0.4	
1748	(*E–E*)-α-farnesene		t	
1773	δ-cadinene		0.2	
1776	γ-cadinene		0.4	
1740	*(Z)-*α*-*bisabolene		t	
1784	(*E*)-α-bisabolene	t	1.1	
1795	geranyl acetate	t		
1808	nerol	0.2		0.3
1849	calamenene		0.2	
1857	geraniol	0.4		0.4
1949	(*Z*)-3-hexenyl nonanoate	t		0.1
2008	caryophyllene oxide	0.2		0.2
**total**		**97.9**	**97.5**	**97.6**

a RRI, relative retention indices calculated against *n*-alkanes.% calculated from FID data; t, trace.

*R. officinalis* essential oil and its fractions determined by GC-FID and GC/MS are listed in Table 3, where the major components 1,8-cineole (47.4%) and camphor (15.2%) were identified.

**Table 3:** Chemical Composition of *R. officinalis* Essential Oil, *n*-Hexane, Diethyl Ether, and Methanol Fractions^a^

		%			
RRI	compound	*R. officinalis* essential oil	*n*-hexane fraction	diethyl ether fraction	methanol fraction
1014	tricyclene	0.4	0.8		
1032	α-pinene	12.0	**28.0**		
1035	α-thujene	t	1.2		
1072	α-fenchene	t	0.3		
1076	camphene	3.5	8.8		
1118	β-pinene	7.1	**20.2**		
1132	sabinene	t	0.1		
1135	thuja-2,4-(10)-diene	t	0.1		
1159	δ-3-carene		0.1		
1174	myrcene	1.2	4.0		
1176	α-phellandrene		0.2		
1183	pseudolimonene		0.1		
1188	α-terpinene	0.3	0.8		
1203	limonene	2.3	8.3		
1218	β-phellandrene		0.7		
1246	(*Z*)-β-ocimene		0.1		
1213	1,8-cineole	**47.4**		**67.4**	
1255	γ*-*terpinene	0.4	1.3		
1266	*(E)-*β-ocimene		0.1		
1280	*p*-cymene	1.7	6.2		
1290	terpinolene	t	0.6		
1452	α-*p*-dimethyl styrene	t	0.1		
1452	1-octen-3-ol	t		0.2	
1466	α-cubebene	t	0.1		
1493	α*-*ylangene		0.1		
1497	α-copaene	t	0.5		
1532	camphor	**15.2**		**23.5**	
1553	linalool	0.9		1.6	3.7
1562	isopinocamphone	t		t	
1586	pinocarvone	t		t	
1589	isocaryophyllene		0.1		
1590	bornyl acetate	0.6		0.9	
1598	camphene hydrate			t	
1611	terpinen-4-ol	0.3		0.4	
1612	β*-*caryophyllene	2.8	**15.4**		
1617	lavandulyl acetate				5.0
1628	aromadendrene		0.1		
1664	*trans*-pinocarveol			0.1	
1682	δ*-*terpineol	0.1			4.5
1687	α-humulene	0.2	1.2		
1704	δ-muurolene		0.3		
1706	α-terpineol	1.6		2.6	
1715	γ-terpineol			0.2	
1719	borneol	1.6		2.7	2.3
1725	verbenone	t		t	
1740	α-muurolene		0.1		
1744	α*-*selinene		t		
1751	carvone				2.6
1773	δ-cadinene	t	0.3		
1776	γ*-*cadinene		0.1		
1799	cadina-1,4-diene		t		
1849	calamenene		0.1		
2008	caryophyllene oxide				**44.6**
2186	eugenol	t			8.1
2239	carvacrol	0.3			
**total**		**99.9**	**100**	**99.6**	**70.8**

a RRI, relative retention indices calculated against *n*-alkanes; the % was calculated from FID data; t, trace.

The *S. sclarea* essential oil components and fractions are listed in Table 4 by more than 65 identified compounds.

**Table 4:** Chemical Composition of *S. sclarea* Essential Oil, *n*-Hexane, Diethyl Ether, and Methanol Fractions^a^

		%			
RRI	compound	*S. sclarea* essential oil	*n*-hexane fraction	diethyl ether fraction	methanol fraction
1032	α-pinene	t	0.2		
1035	α-thujene		t		
1076	camphene		t		
1118	β-pinene	**t**	0.3		
1132	sabinene		0.1		
1174	myrcene	0.5	5.5	0.2	
1176	α-phellandrene	0.2	0.8		
1188	α-terpinene	t	0.1		
1203	limonene	0.4	6.3	t	
1213	1,8-cineole	0.1		t	
1218	β-phellandrene		0.3		
1220	*cis-*anhydrolinalool oxide	t		t	
1222	2-hexanol	t		t	
1246	(*Z*)-β-ocimene	0.1	2.2	t	
1253	*trans*-anhydrolinalool oxide	t		t	
1255	γ-terpinene		0.2		
1266	(*E*)-β-ocimene	0.3	3.8	0.1	
1280	*p*-cymene	0.4	5.0	t	
1290	terpinolene	0.1	1.0	t	
1413	rose furan		0.1		
1429	perillene		0.1		
1466	α-cubebene		0.3		
1497	α-copaene	0.3	5.6		
1528	α-burbonene	0.1	0.2		
1532	camphor	0.3		0.4	
1535	β-bourbonene	0.1	3.1		
1553	linalool	**20.3**		**20.6**	18.6
1568	*trans*-α-bergamotene		t		
1549	β-cubebene	t	1.6		
1565	linalyl acetate	**63.9**	0.3	**65.7**	**44.7**
1579	acetoxylinalool oxide			0.1	
1589	β-ylangene		0.5		
1594	*trans*-β-bergamotene		t		
1597	β-copaene		0.6		
1600	linalyl isobutyrate	t		0.1	
1600	β-elemene	t	1.7		
1611	terpinen-4-ol	0.1		0.1	
1612	β-caryophyllene	1.2	**28.3**	0.1	
1617	lavandulyl acetate				6.7
1628	aromadendrene		0.2		
1669	sesquisabinene		0.2		
1687	α-humulene	t	1.4		
1694	neral			0.1	
1704	γ-muurolene		0.3		
1706	α-terpineol	4.0		4.2	9.9
1726	germacrene D	0.8	**16.2**		
1733	neryl acetate	1.1		1.3	
1740	geranial	t		0.2	
1742	β-selinene		1.1		
1744	α-selinene		0.2		
1748	(*E–E*)-α-farnesene		0.8		
1755	bicyclogermacrene		0.5		
1773	δ-cadinene	t	1.4		
1776	γ-cadinene		0.2		
1795	geranyl acetate	2.0		2.4	
1808	nerol	0.7		0.8	
1849	calamenene		0.1		
1857	geraniol	1.6		1.8	
1864	*p*-cymen-8-ol	t		0.1	
1949	(*Z*)-3-hexenyl nonaoate			0.1	8.5
1961	3,7-dimethyl-1,5-octadiene-3,7-diol	0.2		0.2	
2008	caryophyllene oxide	0.4		0.4	
2144	spathulenol	t		0.1	
2257	β-eudesmol	t		0.1	
2312	9-geranyl *p*-cymene	0.1	2.3	t	
2419	sclareol	0.9			
**total**		**99.8**	**93.1**	**99.1**	**88.4**

a RRI, relative retention indices calculated against *n*-alkanes; the % was calculated from FID data; t, trace.

The composition of *M. chamomilla* essential oil and its fractions was also determined by GC-FID and GC/MS methods, as shown in Table 5.

**Table 5:** Chemical Composition of *M. chamomilla* Essential Oil, *n*-Hexane, Diethyl Ether, and Methanol Fractions^a^

		%			
RRI	compound	*M. chamomilla* essential oil	*n*-hexane fraction	diethyl ether fraction	methanol fraction
1174	myrcene	T	T		
1176	α-phellandrene	T	T		
1203	limonene	T	0.1		
1244	2-pentyl furan	T	T		
1246	(*Z*)-β-ocimene	T	0.1		
1255	γ-terpinene	0.1	0.2		
1266	(*E*)-β-ocimene	0.2	0.5		
1280	*p*-cymene	0.1	0.1		
1358	artemisia ketone	0.2			
1400	nonanal	T			
1403	yomogi alcohol	T			
1495	bicycloelemene	T	0.2		
1497	α-copaene	T	0.2		
1510	artemisia alkol	0.1		0.1	
1550	α-isocomene	0.1	t		
1565	linalyl acetate		0.2		
1600	β-elemene	T	0.3		
1612	β-caryophyllene	T	0.3		
1628	aromadendrene	T	0.2		
1661	alloaromadendrene	0.1	0.3		
1668	(*E*)-β-farnesene	**17.0**	**69.6**	0.1	
1704	γ-muurolene	0.1	0.6		
1708	ledene	0.1	0.4		
1726	germacrene D	1.2	4.3		
1594	trans-β-bergamotene	0.1	0.4		
1755	bicyclogermacrene	0.3	2.8		
1758	(*E*–*E*)-α-farnesene	0.7	3.1		
1773	δ-cadinene	0.1	1.0		
1776	γ-cadinene	0.1	0.6		
1783	β-sesquiphellandrene		0.1		
1786	*ar*-curcumene	T	0.2		
1799	cadina-1,4-diene		0.1		
1807	α-cadinene	t	t		
1941	α-calacorene	0.5	0.7		
1946	dendrolasin		0.6		
2050	*(E)*-nerolidol	0.1		0.1	
2098	globulol	t		0.1	
2131	hexahydrofarnesyl acetone	0.3		0.2	
2144	spathulenol	0.4		0.8	
2156	α-bisabolol oxide B	**5.2**		**7.1**	
2187	*T*-cadinol	0.5		0.9	
2200	α-bisabolon oxide A	5.0		5.9	
2232	α-bisabolol	**19.4**		**25.2**	**34.0**
2298	decanoic acid	0.5		t	
2300	tricosane	0.1	0.9		
2400	tetracosane		0.4		
2430	chamazulene	1.6	6.8		
2438	α-bisabolol oxide A	**41.6**		**56.6**	**66.0**
2400	pentacosane	0.3	2.7		
2500	heptacosane		0.7		
2931	hexadecanoic acid	0.4	t		
**total**		**96.5**	**98.7**	**97.1**	**100**

a RRI, relative retention indices calculated against *n*-alkanes; the % was calculated from FID data; t, trace.

### 2.2 Characterization Studies of Emulsions

**Figure 1:**
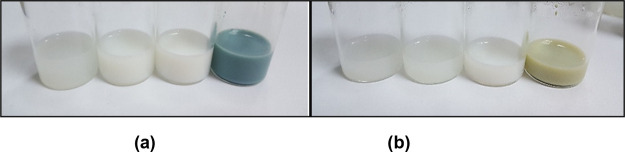
(a) Essential oil emulsions (from left to right): *R. officinalis,S. sclarea,L. latifolia,* and *M. chamomilla.* (b) Emulsions of diethyl ether fractions (from left to right): *R. officinalis,S. sclarea,L. latifolia,* and *M. chamomilla*.

Using the water titration method, emulsions of four essential oils and their diethyl ether fractions were successfully prepared, as shown in Figure 1. The results of characterization studies, including pH, refractive index, turbidity, droplet size, polydispersity index (PDI), and zeta potential values, are listed in Table 6.

**Table 6:** Characterization Results of the Emulsions of Essential Oil and Diethyl Ether Fractions^a^

essential oil emulsion	droplet size (nm ± std)	polydispersity ± std.	zeta potential ± std	refractive index	absorbance (600 nm)	pH	centrifugal resistance	image
*R. officinalis*	40.83 ± 6.50	0.89 ± 0.10	–40.8 ± 2.31	1.3192	0.067	6.2	+	turbid
*R. officinalis* diethyl ether fraction	110.23 ± 27.5	0.69 ± 0.28	–19.4 ± 0.52	1.3192	0.084	5.8	+	milky
*S. sclarea*	114.47 ± 1.19	0.12 ± 0.03	–11.47 ± 0.38	1.3192	0.264	4.08	+	milky
*S. sclarea* diethyl ether fraction	116.97 ± 1.25	0.11 ± 0.005	–8.24 ± 0.37	1.3192	0.232	4.12	+	milky
*L. latifolia*	151.87 ± 4.8	0.26 ± 0.01	–1.08 ± 1.30	1.3190	0.057	5.65	-	milky
*L. latifolia* diethyl ether fraction	266.83 ± 4.2	0.59 ± 0.003	–10.57 ± 0.29	1.3192	0.095	5.43	-	milky
*M. chamomilla*	223.3 ± 0.95	0.20 ± 0.004	–16.8 ± 0.56	1.3194	1.765	5.63	+	blue milky
*M. chamomilla* diethyl ether fraction	214.67 ± 2.4	0.12 ± 0.01	–16.17 ± 0.45	1.3192	0.948	5.52	+	green milky

a +, No change; -, phase separation; std, standard deviation.

According to the results, phase separation and creaming were not observed in the essential oil emulsions evaluated for their centrifugal stability, where the pH of the emulsions ranged between 4.08 and 6.2. The droplet sizes of the emulsions obtained from rosemary essential oil and diethyl ether fraction were measured as 40.83 ± 6.50 and 110.23 ± 27.54 nm, respectively. As an experimental observation, the rosemary essential oil emulsion displayed better results than the diethyl ether fraction emulsion. The droplet size and PDI value of the *S. sclarea* essential oil emulsion were determined as 114.47 ± 1.19 nm and 0.12 ± 0.03, respectively, as seen in Table 6. The droplet size of the emulsion of the *S. sclarea* essential oil diethyl ether fraction was 116.97 ± 1.25 nm, and the PDI value was 0.11 ± 0.005. The droplet sizes of the spike lavender essential oil emulsion and diethyl ether fraction were 151.87 ± 4.76 and 266.83 ± 4.19 nm, respectively. According to the observed results, the essential oil emulsion contains more homogeneous and smaller droplets than the fraction emulsion (see Table 6). As seen in Figure 1, the chamomile essential oil appears blueish milk; its fraction is greenish milk due to its natural color. The absorbance values of 1:100 dilutions are high compared to other tested oils. The droplet sizes (223.3 ± 0.95 and 214.67 ± 2.36 nm, respectively) and PDI values (0.20 ± 0.004 and 0.12 ± 0.01, respectively) of emulsions prepared from chamomile essential oil and diethyl ether fraction were in the same range. Supporting Information presents the droplet size distribution spectrum of the emulsions containing heterogeneous droplets.

In this comparative study, *R. officinalis,S. sclarea*, *L. latifolia,* and *M. chamomilla* essential oils, their emulsions, and diethyl ether fraction emulsions are prepared by the water titration method, with a droplet size of 40.83 ± 6.50 to 266.83 ± 4.19 nm. However, the *S. sclarea* and *M. chamomilla* essential oils and diethyl ether fraction emulsion PDI values 0.11 ± 0.005–0.20 ± 0.004 were lower when compared to those of *R. officinalis* and *M. chamomilla* emulsions. Large droplets and low zeta potential values were determined; also, phase separation after centrifugation was observed only in spike lavender essential oil and its diethyl ether fraction emulsions.

Overall, the refractive index of the prepared emulsions in the presence of essential oil ranged between 1.3190 and 1.3194, suggesting no significant difference between the refractive index values of the formed emulsions.

### 2.3 Antimicrobial Activity

According to the results given in Table 7, the best inhibitory effect was observed in the *S. sclarea* diethyl ether fraction and *M. chamomilla* essential oil at a concentration of 1.25 mg/mL against *S. aureus*.

**Table 7:** MIC Values of Essential Oils, *n*-Hexane, and Diethyl Ether Fractions, Selected Active Components (mg/mL), and Standard Antimicrobials (μg/mL) Determined by the Microdilution Method against *S. aureus*

samples	MIC^a^
*R. officinalis* essential oil	2.5
*R. officinalis n-*hexane fraction	>5
*R. officinalis* diethyl ether fraction	>5
*S. sclarea* essential oil	>5
*S. sclarea n-*hexane fraction	>5
*S. sclarea* diethyl ether fraction	**1.25**
*L. latifolia* essential oil	5
*L. latifolia n-*hexane fraction	>5
*L. latifolia* diethyl ether fraction	>5
*M. chamomilla* essential oil	**1.25**
*M. chamomilla n-*hexane fraction	>5
*M. chamomilla* diethyl ether fraction	2.5
1,8-cineole	>5
linalool	>5
linalyl acetate	5
borneol	**1.25**
bornyl acetate	**0.08**
α-terpineol	2.5
(-)-bisabolol	**0.16**
farnesene isomers	>5
**amoxicillin/clavulanic acid**	**1**
**potassium clavulanate**	**8**
**cefuroxime**	**16**
**chloramphenicol**	**4**
**% DMSO**	**12.5**

a MIC, minimum inhibitory concentration.

In this study, the in vitro antimicrobial effects of *R. officinalis* leaf essential oils [1,8-cineole (47.2–27.5%) and camphor (12.9–27.9%)] collected from different regions were evaluated. MIC values of 5–1, 25 μL/mL were obtained against *S. aureus* ATCC 6538.^26^ In a study investigating the antimicrobial effect of rosemary essential oil,^15^ an effect against *S. aureus* clinical isolate was observed at a concentration of 0.03 v/v. In this study, 1,8-cineole (26.54%), the main component of the essential oil, was effective at 0.3 v/v. Similarly, in our study, rosemary essential oil (MIC: 2.5 mg/mL) was more effective than the main component 1,8-cineole (MIC: >5) against standard *S. aureus* strains. In another study, the agar dilution method evaluated the antimicrobial activity of rosemary essential oil, and essential oil was found to be effective against *S. aureus* ATCC 25923 at 6.40 mg/mL.^27^ In the study of Mekonnen et al., the main components of *R. officinalis* essential oil were determined as 50.83% α-pinene and 24.42% 1,8-cineole. In this study, the antimicrobial effect of essential oil (50 μL) against the *S. aureus* strain was assessed by the agar diffusion method, an inhibition zone of 25 ± 1.23 mm was observed, and the MIC value was determined as 23.25 mg/mL.^28^

In another study, the agar dilution method evaluated the antimicrobial activity of *S. sclarea* essential oil. Accordingly, the essential oil was effective at 0.29 mg/mL against *S. aureus* ATCC 25923.^27^ The antimicrobial effect of the essential oil containing 12.7% linalyl acetate, 21.1% α-terpinyl acetate, and 47.4% α-terpineol was assessed against *S. aureus* ATCC 25923. *S. sclarea* oil showed a microbiostatic effect (MIC: 1.5–2.0 mg/mL) against *S. aureus* and *S. epidermidis* strains.^23^

It was reported that 50 μL of *M. chamomilla* essential oil, which contained α-bisabolol oxide B (51.4%) and chamazulene/azulene (17.7%), was inefficient against *S. aureus*.^28^ Chamomile essential oil showed a 21 mm inhibition zone against *S. aureus* ATCC 9244 in the disk diffusion method at a concentration of 30 mg/mL. The MIC values obtained in this study were 2 μg/mL against the same strains.^29^ In the disk diffusion method of essential oil (1 μg/mL) containing 43.5% trans-β-farnacene, an inhibition zone diameter of 10 mm was obtained against the *S. aureus* strain. In this study, MIC values obtained against the *S. aureus* strain by the microdilution method were 8 μg/mL, respectively.^30^

A previous study determined the antibacterial effect of the *L. latifolia* essential oil against *S. aureus* and *L. monocytogenes* strains in the 0.5–2 μL/mL concentration range.^31^ In our previous study,^32^ the effect of the *L. latifolia* essential oil against the *S. aureus* ATCC 25923 strain was determined as 2.5 mg/mL by microdilution. This study combined the essential oil and the camphor using the checkerboard method. As a result, combinations with synergistic and additive effects were determined.

According to Table 8, the best inhibitory effect was observed for chamomile essential oil emulsion and diethyl ether fraction emulsion at a concentration of 0.098 mg/mL. In addition, the rosemary essential oil emulsion showed a significant antimicrobial effect at a concentration of 0.78 mg/mL and diethyl ether fraction emulsion at 1.56 mg/mL, respectively. Emulsions prepared with clary sage essential oil and its diethyl ether fraction showed a MIC value of 0.78 mg/mL. Emulsions prepared with spike lavender essential oil and diethyl ether fraction showed MIC values of 0.39 and 0.78 mg/mL, respectively. The observed results were relatively more active compared to the antimicrobial effects of the tested essential oils. According to experimental data, it can be concluded that the antibacterial effect was increased with the emulsification of chamomile essential oil.

**Table 8:** Antibacterial (MIC) and Anti-Biofilm (MBIC) Effects of Essential Oil Emulsions against the *S. aureus* Strain (mg/mL)^a^

samples	antibacterial effect (mg/mL)	anti-biofilm effect (mg/mL)
*R. officinalis*	0.78	0.78
*S. sclarea*	0.78	
*L. latifolia*	0.39	
*M. chamomilla*	0.098	0.02
*L. latifolia* diethyl ether fraction	0.78	
*R. officinalis* diethyl ether fraction	1.56	1.56
*S. sclarea* diethyl ether fraction	0.78	
*M. chamomilla* diethyl ether fraction	0.098	0.05
**amoxicillin/clavulanate (μg/mL)**	**1**	**2**

a MIC, minimum inhibitory concentration; MBIC, minimum biofilm inhibitory concentration.

This study present determined the anti-biofilm effect by incubating the *S. aureus* strain in a 96-well flat bottom plate for 48 h to form a biofilm. Table 8 presents the results of tested emulsions against *S. aureus*. Accordingly, the anti-biofilm effect of emulsions obtained with sage essential oil and its diethyl ether fraction, spike lavender, and the diethyl ether fraction of this oil was not susceptible within the working concentration ranges (0.01–10 mg/mL). Remarkable anti-biofilm effects were observed in chamomile essential oil and diethyl ether fraction at a concentration of 0.02 and 0.05 mg/mL, respectively.

In vitro antibacterial activity tests of essential oils and their respective emulsions against the sample pathogen *S. aureus* were performed. According to the results, essential oils, *n*-hexane, and diethyl ether fractions showed antimicrobial effects at the 1.25–5 < mg/mL concentration range, as reported in Table 7. The antimicrobial effective concentration range of the essential oil and diethyl ether fraction emulsions was 0.098–1.56 mg/mL, where the details are provided in Table 8. The cumulative results showed that the antibacterial effect was increased by emulsification of the chamomile essential oil.

In addition, the anti-biofilm effect results of essential oil emulsions and fraction emulsions against *S. aureus* were in the same range (Table 8). Thus, rosemary and chamomile essential oils and their fractions showed an anti-biofilm effect. However, this effect was not observed in the studied concentration range of 0.01–10 mg/mL in clary sage and spike lavender essential oils.

### 2.4 Synergistic Antibacterial Effect Results from Essential Oil Combinations

The synergistic antibacterial effects of the binary combinations 10:90, 20:80, 30:70, 40:60, 50:50, 60:40, 70:30, 80:20, and 90:10 (v:v) of emulsions were determined. As a result, fractional inhibitory concentration (FIC) values were determined over a wide concentration range by combining the samples at 72 different concentrations. According to the values provided in Table 9, 21 different emulsion combinations were synergistic (FIC ≤ 0.5), whereas other combinations were concluded as additive (0.5 < FIC ≤ 1) or ineffective (1 < FIC < 4).

**Table 9:** FICs of Essential Oil Emulsion Combinations^a^

	combinations (v/v)								
samples	10:90	20:80	30:70	40:60	50:50	60:40	70:30	80:20	90:10
*R. officinalis* + *S. sclarea*	0.95	**0.45**	**0.42**	**0.40**	**0.36**	**0.35**	0.65	0.60	1.11
*R. officinalis* + *L. latifolia*	1.11	**0.3**	**0.33**	**0.35**	**0.36**	**0.40**	**0.42**	**0.45**	**0.47**
*R. officinalis* + *M. chamomilla*	-	-	-	-	-	-	-	-	-
*R. officinalis* + *L. latifolia* fraction	1.11	0.6	0.65	0.70	**0.37**	**0.4**	**0.42**	**0.45**	**0.47**
*R. officinalis* + *R. officinalis* fraction	-	-	-	-	-	-	-	-	-
*R. officinalis* + *S. sclarea* fraction	1	1	1	1	1	1	1	1	1
*R. officinalis* + *M. chamomilla* fraction	-	-	-	-	-	-	-	-	-
*S. sclarea* + *L. latifolia*	1.10	0.60	0.65	0.70	0.73	0.80	0.85	0.90	0.99
*S. sclarea* + *M. chamomilla*	1.15	0.63	1.33	1.43	0.74	0.80	0.85	0.90	0.95
*S. sclarea* + *L. latifolia* fraction	1.31	0.81	0.94	1.10	0.61	0.70	0.77	0.85	0.92
*S. sclarea* + *R. officinalis* fraction	1.24	1.00	0.68	0.87	1.06	0.63	0.72	0.81	0.90
*S. sclarea* + *S. sclarea* fraction	1.10	1.20	0.65	0.70	0.73	0.80	0.85	0.90	0.95
*S. sclarea* + *M. chamomilla* fraction	1.15	1.25	1.33	1.43	1.51	1.62	0.85	1.80	0.95
*L. latifolia* + *M. chamomilla*	1	0.95	0.88	0.83	0.76	0.71	0.67	1.22	1.11
*L. latifolia* + *L. latifolia* fraction	1.11	1.20	0.65	0.70	0.75	0.80	0.85	0.90	0.95
*L. latifolia* + *R. officinalis* fraction	1	0.80	0.95	**0.55**	0.63	0.70	0.40	0.85	0.93
*L. latifolia* + *S. sclarea* fraction	1	1	**0.5**	1	1	1	1	1	1
*L. latifolia* + *M. chamomilla* fraction	1	0.94	0.88	0.83	0.76	0.71	0.67	0.61	**0.55**
*M. chamomilla* + *L. latifolia* fraction	0.66	0.82	0.98	1.14	0.63	0.73	0.81	0.89	0.97
*M. chamomilla* + *R. officinalis* fraction	-	-	-	-	-	-	-	-	-
*M. chamomilla* + *S. sclarea* fraction	1	1	1	1	1	2	1	1.05	1.05
*M. chamomilla* + *M. chamomilla* fraction	1	1	1	1	1	1	1	1	1

a -, Not detected.

Synergistic effects were observed for the combination of rosemary and clary sage essential oil emulsions at 20:80, 30:70, 40:60, 50:50, and 60:40 proportional ratios. Synergistic effects were observed in all rosemary and spike lavender essential oil emulsion combinations except at the 10:90 ratio. In addition, a synergistic effect of the 50:50, 60:40, 70:30, 80:20, and 90:10 emulsion ratios of the diethyl ether fractions of rosemary and spike lavender essential oils was observed. The results of rosemary essential oil emulsion combinations with chamomile essential oil emulsion, chamomile diethyl ether fraction emulsions, and rosemary essential oil diethyl ether fraction emulsion were, however, not observed. The combinations of rosemary essential oil emulsion and clary sage diethyl ether fraction emulsions showed interestingly additive results. Among the clary sage essential oil emulsions, only the combinations with rosemary essential oil showed synergistic effects. Synergistic concentrations were observed in spike lavender essential oil, rosemary essential oil, and diethyl ether fraction emulsion combinations. In addition, a synergistic antibacterial effect was achieved by combining spike lavender essential oil emulsion and clary sage diethyl ether fraction emulsion at a 30:70 ratio. Also, a spike lavender oil and chamomile diethyl ether fraction emulsion in a ratio of 90:10 showed similar inhibitory effects. The chamomile oil and rosemary essential oil diethyl ether fraction combination emulsions were unsuccessful due to solubility issues. The synergistic effect was obtained from combining this oil with diethyl ether fraction emulsion combinations only by the spike lavender essential oil emulsion. At other combination concentrations, additive or ineffective combinations were classified (Table 9).

Experimental studies for the enhancement of the targeted antimicrobial effects by combining various essential oils were reported in previous studies.^33−35^ Recently, studies were conducted on different biological activity tests using essential oil nanoemulsions rather than pure oil. In this study, 72 concentrations were produced from each combination, and FIC values were determined in a wide concentration range. Twenty-one emulsion combinations had synergistic effects according to FIC values.

Although there are numerous antimicrobial evaluation reports on sage, spike lavender, rosemary, and chamomile essential oils,^15−24^ to the best of our knowledge, this is the first experimental study on the antimicrobial combinations of the essential oils as well as their different fractions obtained.

### 2.5 Anti-Inflammatory Effect Results

The anti-inflammatory potential of the same four essential oils, their *n*-hexane and diethyl ether fractions, and their emulsions were evaluated using the in vitro 5-lipoxygenase (5-LOX) inhibition assay. According to the results represented in Table 10, the highest inhibitory percentage was observed in the rosemary essential oil diethyl ether fraction with a value of 43.5 ± 2.9% and its respective emulsion with 63.9 ± 0.6%. As expected, the relative percentages of active components in each fraction of rosemary essential oil increased by fractionation. The main reason for the increase of the anti-inflammatory effect by fractionation can be linked to the 47.4% relative amount of 1,8-cineole, while the diethyl ether fraction contained 67.4% enrichment. The anti-inflammatory effect of clary sage essential oil at 50 μg/mL concentration showed 23.2 ± 3.9% LOX inhibition. The main component percentages of this oil in *n*-hexane and diethyl ether fractions were 63.9% linalyl acetate, 28.3% β-caryophyllene, and 65.7% linalyl acetate, respectively. The anti-inflammatory effects of the clary sage essential oil and diethyl ether fraction were in the same range due to the main component and relative content similarities. The emulsions of clary sage oil (28.6 ± 1.5%) and diethyl ether fraction (27.5 ± 0.2%) were slightly more effective than the initial oil.

**Table 10:** Results of Anti-Inflammatory Effects of Essential Oils, Fractions, and Emulsions^a^

samples (50 μg/mL)	% inhibition ± std
rosemary essential oil	17.2 ± 3.4
rosemary essential oils *n*-hexane fraction	5.7 ± 1.8
rosemary essential oil diethyl ether fraction	**43.5 ± 2.9**
rosemary essential oil emulsion	17.4 ± 1.3
rosemary essential oil diethyl ether fraction emulsion	**63.9 ± 0.6**
clary sage essential oil	23.2 ± 3.9
clary sage essential oil *n*-hexane fraction	18.8 ± 3.1
clary sage essential oil diethyl ether fraction	18.6 ± 1.5
clary sage essential oil emulsion	28.6 ± 1.5
clary sage essential oil diethyl ether fraction emulsion	27.5 ± 0.2
lavender essential oil	32.7 ± 2.5
lavender essential oil *n*-hexane fraction	10.3 ± 1.4
lavender essential oil diethyl ether fraction	15.5 ± 4.3
lavender essential oil emulsion	18.1 ± 2.4
lavender essential oil diethyl ether fraction emulsion	21.1 ± 4.7
chamomile essential oil	16.3 ± 0.5
chamomile essential oil *n*-hexane fraction	20.0 ± 3.1
chamomile essential oil diethyl ether fraction	22.9 ± 0.7
chamomile essential oil emulsion	10.2 ± 2.5
chamomile essential oil diethyl ether fraction emulsion	23.0 ± 0.6
**NDGA (control)**	100 (IC_50_: 2.95 ± 0.21 μg/mL)

a std, standard deviation.

The results of the anti-inflammatory effect of spike lavender essential oil, fractions, and their related emulsions are listed comparatively in Table 10. Accordingly, the LOX inhibition of spike lavender essential oil, *n*-hexane fraction, diethyl ether fraction, the emulsion of essential oil, and diethyl ether fraction emulsion was 32.7 ± 2.5, 10.3 ± 1.4, 15 ± 4.3, 18.1 ± 2.4, and 21.1 ± 4.7%, respectively. Overall, the inhibitory effect results of the spike lavender samples after emulsifications were limited.

LOX inhibition of chamomile essential oil, *n*-hexane fraction, diethyl ether fraction, essential oil emulsion, and diethyl ether fraction emulsion at 50 μg/mL was 16.3 ± 0.5, 20 ± 3.1, 22.9 ± 0.7, 10.2 ± 2.5, and 23 ± 0.6%, respectively. The analyzed chamomile oil contained 19.4% α-bisabolol and 41.6%, whereas the diethyl ether fraction had 25.2% α-bisabolol and 56.6% α-bisabolol oxide A*,* respectively. The anti-inflammatory effects of the diethyl ether fraction and the diethyl ether fraction emulsion also relatively increased compared to the oil due to the enriched chemical composition.

In the present study, the anti-inflammatory effects of essential oils, their respective *n*-hexane, diethyl ether fractions, and their emulsions were determined by in vitro LOX inhibition, where essential oil emulsification resulted in enhanced anti-inflammatory effects.

### 2.6 Antioxidant Effect Results

In this study, three different methods were used to determine in vitro antioxidant potential and capacity. The results obtained were evaluated comparatively, as illustrated in the following sections.

#### 2.6.1 DPPH (1,1-Diphenyl-2-picrylhydrazyl) Radical Scavenging Effect

The percentage of antioxidant effects of essential oils, fractions, and their respective emulsions determined by this method at 0.5 mg/mL are presented in Table 11. The antioxidant effects of sage and spike lavender samples at concentrations of 0.5–0.001 mg/mL were insignificant.

**Table 11:** DPPH Radical Scavenging, ABTS Radical Anion Scavenging, and Cupric Reducing Antioxidant Capacity (CUPRAC Test) of Essential Oils, Fractions, and Emulsions^a^

	% inhibition ± std		
samples	DPPH (0.5 mg/mL)	ABTS (0.4 mg/mL)	CUPRAC (0.3 mg/mL)
rosemary essential oil	14.2 ± 0.7	-	7.8 ± 2.7
rosemary essential oil *n*-hexane fraction	-	-	-
rosemary essential oil diethyl ether fraction	-	-	-
rosemary essential oil emulsion	8.8 ± 2.8	10.0 ± 3.3	14.9 ± 4.6
rosemary essential oil diethyl ether fraction emulsion	21.5 ± 0.5	**34.3 ± 4.4**	27.2 ± 5.1
clary sage essential oil	-	-	-
clary sage essential oil *n*-hexane fraction	-	-	-
clary sage essential oil diethyl ether fraction	-	-	-
clary sage essential oil emulsion	-	-	-
clary sage essential oil diethyl ether fraction emulsion	-	-	-
lavender essential oil	6.3 ± 0.9	6.4 ± 2.3	-
lavender essential oil *n*-hexane fraction	-	-	-
lavender essential oil diethyl ether fraction	-	-	-
lavender essential oil emulsion	4.0 ± 1.2	5.8 ± 1.0	1.9 ± 0.4
lavender essential oil diethyl ether fraction emulsion	3.5 ± 0.7	3.8 ± 0.3	-
chamomile essential oil	**56.4 ± 0.7 (EC_50_: 0.41 ± 0.00)**	**42.4 ± 1.0**	10.4 ± 3.8
chamomile essential oil *n*-hexane fraction	**46.2 ± 2.0**	**35.4 ± 5.3**	5.5 ± 1.9
chamomile essential oil diethyl ether fraction	18.8 ± 1.7	12.9 ± 3.9	15.6 ± 4.4
chamomile essential oil emulsion	**53.7 ± 1.6 (EC_50_: 0.38 ± 0.01)**	23.9 ± 6.3	**42.8 ± 2.7**
chamomile essential oil diethyl ether fraction emulsion	15.3 ± 0.8	25.5 ± 2.6	26.3 ± 2.4
**ascorbic acid** (0.02 mg/mL)	59.1 ± 0.8 (EC_50_: 0.008 ± 0.001)	83.8 ± 3.8 (EC_50_: 0.01 ± 0.001)	EC_50_: 0.02 ± 0.00
**gallic acid** (0.004 mg/mL)	58.0 ± 0.9 (EC_50_: 0.002 ± 0)		EC_50_: 0.01 ± 0.00

a -, no results were obtained due to turbidity/low activity; std, standard deviation.

The DPPH radical scavenging effect of rosemary essential oil, emulsion, and diethyl ether fraction emulsion was determined as 14.2 ± 0.7, 8.8 ± 2.8, and 21.5 ± 0.5%, respectively. The *n*-hexane and diethyl ether fraction results could not be observed due to experimental conditions. It is proposed that due to the high 1.8-cineole content in the diethyl ether fraction and emulsification, this sample may have a higher antioxidant effect than the others. In the study of Zaouali et al., the impact of antioxidant (EC_50_) of rosemary essential oil was between 6 and 28 μL/mL by the DPPH method.^26^

Table 11 presents the DPPH radical scavenging effect of chamomile essential oil, *n*-hexane fraction, diethyl ether fraction, the oil emulsion, and diethyl ether fraction emulsion. At a concentration of 0.5 mg/mL, chamomile essential oil showed 56.4 ± 0.7% inhibition, and this oil emulsion was 53.7 ± 1.6%, which is slightly less. In addition, the diethyl ether fraction of this oil showed an 18.8 ± 1.7% inhibition effect, and the diethyl ether fraction emulsion showed 15.3 ± 0.8%, respectively, following the pattern. According to this result, it is suggested that the radical scavenging effect does not change by emulsification. In addition, the *n*-hexane fraction of chamomile essential oil showed an antioxidant effect of 46.2 ± 2.0%. GC/MS results showed that the main active constituent of the *n*-hexane fraction was 69.6% (*E*)-β-farnesene. This component is 17% in essential oil and 0.1% in diethyl ether fraction of this oil, suggesting limited association. In one study, *M. chamomilla* essential oil containing 27.5% chamazulene and 29.4% α-bisabolol oxide A showed 75.6% inhibition in the DPPH method at a concentration of 400 μg/mL.^5^

#### 2.6.2 ABTS [2,2′-Azino-bis(3-ethylbenzothiazoline-6-sulfonic acid)] Radical Anion Scavenging Effect

Table 11 presents the in vitro antioxidant effect of essential oils, fractions, and emulsions at a concentration of 0.4 mg/mL. The experimental results showed that the rosemary oil and its fractions were ineffective, whereas its emulsion showed a weak antioxidant effect with 10.0 ± 3.3%, and the emulsion of the diethyl ether fraction was increased to 34.3 ± 4.4%. Accordingly, it can be hypothesized that the increased solubility of rosemary oil by emulsification may have contributed to enhancing the antioxidant effect.

Interestingly, experimental results of clary sage and spike lavender essential oils, fractions, and emulsions were not remarkable regarding the antioxidant activity.

Chamomile essential oil, *n*-hexane fraction, and diethyl ether fraction showed 42.4 ± 1.0, 35.4 ± 5.3, and 12.9 ± 3.9% ABTS radical scavenging effects, respectively. The emulsion of this oil showed a slight increase in antioxidant effect with 23.9 ± 6.3%, and the emulsion of the diethyl ether fraction showed 25.5 ± 2.6% inhibition. Accordingly, it was suggested that the effect increased with the emulsification of the fraction. However, it was observed that the impact was not raised as expected with the emulsification of the essential oil. The relatively high antioxidant effect of the *n*-hexane fraction was proposed due to the content of the main component (*E*)-β-farnesene, i.e., 69.6%.

#### 2.6.3 CUPric Reducing Antioxidant Capacity (CUPRAC) Results

The results obtained with this method were based on the measurement of the absorbance at 450 nm of the reduction of copper(II)-neocuproine complex to copper(I)-neocuproine, as shown in Table 11. Experimental results from the clary sage and spike lavender essential oils, as well as their fractions at a working concentration of 0.3 mg/mL, were not remarkable.

Rosemary essential oil, emulsion, and its diethyl ether fraction emulsion showed an increased activity trend with 7.8 ± 2.7, 14.9 ± 4.6, and 27.2 ± 5.1% copper reduction power, respectively. In contrast, the *n*-hexane and diethyl ether fractions were relatively low. As a result, it was observed that the effect increased approximately 2-fold by emulsification. In addition, increasing the solubility of the diethyl ether fraction by emulsification and the relatively high percentage of 1,8-cineole contributed to higher antioxidant effects than other tested samples.

The chamomile essential oil CUPRAC test inhibition percentages are listed in Table 11. Accordingly, the oil, its *n*-hexane, and diethyl ether fractions showed 10.4 ± 3.8, 5.5 ± 1.9, and 15.6 ± 4.4% in vitro antioxidant effects, respectively. The antioxidant effects of this oil emulsion and diethyl ether emulsions were 42.8 ± 2.7 and 26.3 ± 2.4%, respectively. As a result, the consequence increased by essential oil emulsification and its fractions with the aid of this method.

Overall, in vitro antioxidant results obtained by three different methods showed that the essential oils, fractions, and emulsions influenced the activity. Thus, the variations of the antioxidant effect showed differences according to the methods. The antioxidant effect was increased by rosemary oil fractionation and emulsification. The antioxidant activity of chamomile oil increased by fractionation and emulsification, however, only in ABTS and CUPRAC methods. Interestingly, the *n*-hexane fraction of chamomile essential oil was highly effective in DPPH and ABTS methods, while it was less effective in the CUPRAC method used. These methodological differences are due to variations in the polarity of the tested individual samples, which were incompatible with the respective test method. Additionally, solubility problems of essential oils and turbidity were limiting factors at high concentrations.

In vitro antioxidant effects of the samples were evaluated using three different methods (DPPH, ABTS, and CUPRAC), and the results were assessed comparatively, where the chamomile essential oil showed the best activity ranges. It was observed that the antioxidant effect was increased by fractionation and emulsification. The mechanism for such an observation could be enhanced solubility of emulsions containing nano-sized droplets.

## 3 Conclusions

The findings of this study, for the first time, highlighted the in vitro synergistic antibacterial and antibiofilm activity as well as the antioxidant and anti-inflammatory activity of essential oil emulsions. Based on the experimental observations, essential oil and fraction combinations enhanced the solubility by nanoemulsions, which opened new insights into known biological activities. However, more detailed advanced experimental studies, such as in vivo biological activity tests on animal models, are still needed.

## 4 Materials and Methods

### 4.1 Essential Oils and Fractionation by Vacuum-Column Chromatography

Pharma-grade essential oils of *Lavandula latifolia* Medik. (Apoth. Bauer & Co., Germany), *Matricaria chamomilla* L. (from Caesar & Loretz), *Rosmarinus officinalis* L. (Caesar & Loretz, Germany), and *Salvia sclarea* L. (from Apoth. Bauer & Co., Germany) were obtained from commercial sources. Samples were also stored in the depository at Anadolu University, Faculty of Pharmacy, Pharmacognosy Department, Turkey.

For the essential oils’ fractionation, vacuum-column chromatography was used.^36^ Silica gel 60 (Meck-7734, 0.06–0.2 mm) was used as the chromatographic adsorbent. 60 g of silica gel was allowed to activate for 2 h at 100 °C. The silica gel was mixed with *n*-hexane and introduced into the column (1.5 × 50 cm) at room temperature. 6 g of essential oil was fractionated initially using *n*-hexane. After that, using diethyl ether, methanol fractions were collected, respectively. The fractionation process was followed and documented by TLC. The amounts of the fractions are indicated in Table 1. The chemical composition of three different fractions of the four essential oils was characterized by GC-FID and GC/MS, respectively (Tables 2^3^,4,5).

### 4.2 Chromatographic Analysis

#### 4.2.1 Thin Layer Chromatography (TLC)

For the separations, 0.2/0.25 mm silica-coated plates (Merck, Germany) on aluminum support were used, if not otherwise stated. 20 μL of essential oils was dissolved in 1 mL of toluene and applied as 15 μL to the plate.^37^ A toluene-ethyl acetate solvent system (95:5) was developed, which separated the samples, initially visualized by UV, followed by a derivatization reagent (anisaldehyde/sulfuric acid) after 100–105 °C heat application on the TLC plate for 10 min. Results were compared with standards provided within the Supporting Information.

#### 4.2.2 GC/MS Analysis

The analyses were conducted using the Agilent 5975 GC-MSD system. A 60 m × 0.25 mm, 0.25 mm film thick column (Innowax FSC) was utilized in this study. Helium was used as the carrier gas with a rate of 0.8 mL/min. The GC oven’s temperature was kept at 60 °C for 10 min and increased at a rate of 4 °C/min to reach 220 °C. The temperature was kept constant at 220 °C for 10 min and then increased at a rate of 1 °C/min to reach 240 °C. The split ratio and injector temperature were 40:1 and 250 °C, respectively. Mass spectra were recorded at 70 eV, where the mass range was *m/z* 35–450.^38^

#### 4.2.3 GC-FID Analysis

The GC analyses were conducted using an Agilent 6890N GC system. FID (flame ionization detector) temperature was set to 300 °C. Simultaneous auto-injection was performed on the duplicate column of the same operational conditions to obtain the same elution order with the GC/MS system. Relative percentages (%) of the separated compounds were assessed from respective FID chromatograms.^38^

#### 4.2.4 Identification of Components

Essential oil components’ identification was conducted by evaluating the relative retention times (RTs) with those of authentic samples or by comparing the relative retention index (RRI) with a series of *n*-alkanes. Computer matching (Wiley GC/MS Library, MassFinder Software 4.0)^39,40^ and in-house ″Baser Library of Essential Oil Constituents″ libraries developed by genuine compounds and known components were also performed.

### 4.3 Preparation and Characterization of Essential Oil Emulsions

An aliquot of 10% (w/v) essential oil and 10% (w/v) Tween 80 were stirred at 840 rpm in a multi-magnetic stirrer for 15 min. Distilled water (80%) was dropped onto the oil phase at a rate of 0.8 mL per minute with a peristaltic pump, and stirring was continued for a further 30 min.^41^ The resulting emulsions contain 100 mg/mL essential oil.

The formulations were evaluated for phase separation, creaming, and turbidity and incubated at room temperature for 48 h. After that, the mechanical strength test was performed by centrifugation. Besides, droplet size, PDI, and zeta potential values were measured. The emulsions were diluted with distilled water before use in the characterization and bioactivity studies.

#### 4.3.1 Turbidity

The turbidity properties of the emulsions were measured at 600 nm using a UV–visible spectrophotometer (Shimadzu, UV-PharmaSpec 1700) against blank (distilled water) following the method.^42^

#### 4.3.2 Centrifugation

The prepared emulsion systems were centrifuged at 3000 rpm for 20 min by a microcentrifuge (Labnet 24D, USA). Following the centrifugation, the formulations were evaluated for phase separation.^42^ The emulsions undergoing the centrifugation test were diluted 1/100 (v/v), and the following changes in appearance were recorded, if any.

#### 4.3.3 pH

A digital pH meter (Heidolph, Germany) was used to measure the pH values of the formulations after the respective calibrations.

#### 4.3.4 Droplet Size and Polydispersity Index

Prepared emulsions were diluted 1:100 with distilled water for the droplet size, PDI, and zeta potential analyses by the Zeta sizer Nano-ZS (Malvern Instruments, UK) instrument at room temperature.^41^ The refractive index of emulsions was measured by a refractometer (Krüss, Germany) to determine the droplet size. The spectra of the zeta size analyses are given in the Supporting Information.

### 4.4 Synergistic Antibacterial Activity Evaluation by the Microdilution Method

*S. aureus* ATCC 11632 was used as the standard bacterial strain, where Mueller–Hinton agar (MHA) and Mueller–Hinton broth (MHB) were used for the incubation at 37 °C for 24 h. The bacterial density was adjusted according to McFarland No: 0.5 (approx. 10^8^ CFU/mL) by using a turbidimeter.^43,44^

100 μL of the test essential oils and their diethyl ether fraction emulsions were transferred to a 96-well flat-bottom microtiter plate format. Serial dilutions (0.01–10 mg/mL) of the samples were prepared to obtain the resulting concentrations. The density-adjusted microorganisms (100 μL solution diluted to 1%) were added to plates. After 24 h of incubation at 37 °C, 20 μL of 0.01% resazurin solution was added and incubated at 37 °C for 3 h.^45^ All experiments were repeated in triplicate, and average minimum inhibition concentrations (MICs) were reported using positive and negative controls as in Tables 7 and 8.

As given in previous work,^32^ essential oils and diethyl ether fraction emulsions at a concentration of at least 4-fold the MIC values were combined in binary combinations and nine different ratios: 10:90, 20:80, 30:70, 40:60, 50:50, 60:40, 70:30, 80:20, and 90:10 (v/v), respectively. The results are given as fractional inhibition concentration [FIC (A + B) = (MIC AB/MIC A) + (MIC AB/MIC B)] as shown in Table 9. As defined in the literature,^46^ the calculation of FIC ≤ 0.5 was classified as synergistic, 0.5 < FIC ≤ 1 as additive, 1 < FIC < 4 as ineffective, and FIC ≥ 4 as antagonistic.

### 4.5 Determination of the Anti-Biofilm Effect

Bacteria grown as described above were 1/10 diluted, and 200 μL solutions were added into wells. After 48 h of incubation at 37 °C, using a NaCl solution, washing was conducted twice. 100 μL of the test medium and test samples was added to the resulting biofilm layer.^47^ After 24 h of incubation at 37 °C, 20 μL of resazurin was added.^48^ The minimum biofilm inhibition concentrations were determined after triplicate experimentation, and the resulting mean values are listed in Table 8.

### 4.6 In Vitro Anti-Inflammatory Activity: LOX Enzyme Activity Inhibition

LOX (1.13.11.12, 7.9 units/mg) enzyme activity inhibition levels were determined in a special 96-well quartz plate (Hellma), spectrophotometrically (BioTek Synergy, USA) according to previous methods.^45,49^ 1.94 mL of potassium phosphate buffer (100 mM; pH: 8.80), 40 μL of different concentrations of test samples, and 20 μL of LOX were mixed and incubated at 25 °C for 10 min, followed by the addition of 300 μL of this mixture to each well. Subsequently, 7.5 μL of the linoleic acid was added and mixed for 30 s. Nordihydroguaiaretic acid (NDGA) was used as a positive control. Changes in absorbance were measured at 243 nm for 10 min at ten intervals. These experiments were performed four replications, and the results were expressed as inhibition percentage (%*I*).
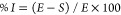
*E*, Enzyme absorbance without sample addition; *S*, Enzyme absorbance with sample addition.

### 4.7 In Vitro Antioxidant Activity Evaluations

#### 4.7.1 DPPH Radical Scavenging Assay

The samples were prepared according to previously described methods^50^ in the 0.5–0.001 mg/mL range. Accordingly, 100 μL of 80 μg/mL DPPH solution was added to the samples for the initiation of the reaction, which was then incubated at room temperature in the dark for 60 min. At the end of the incubation period, UV absorbance values were recorded at 517 nm. Ascorbic acid and gallic acid (0.125–0.0001 mg/mL) were used as positive controls. The experiments were conducted in triplicates, and the percent inhibitions (%*I*) are given in Table 11.

#### 4.7.2 ABTS Radical Anion Scavenging Assay

The assay was performed according to previous work.^51^ The solutions of 7 mM ABTS prepared with 0.1 M phosphate buffer (pH 7.4) and 2.45 mM sodium persulfate (Na_2_S_2_O_8_) were mixed in a 1:2 ratio and incubated in the shaker for 12 h in the dark. The absorbance of the ABTS solution at 734 nm was 0.700 ± 0.025 nm. Afterward, 150 μL of the sample was added to 30 μL of ABTS solution and allowed to incubate at room temperature in the dark for 30 min. Ascorbic acid was used as a standard for comparison, where absorbance values were read at 734 nm. All experiments were repeated in triplicates, and percent inhibition (%*I*) results are listed in Table 11.

#### 4.7.3 CUPRAC Assay

Following the previously reported procedure,^51^ 50 μL of CuCl_2_ solution (0.01 M), 50 μL of ammonium acetate buffer (pH: 7), and 50 μL of neocuproine solution (7.5 × 10^–3^ M) were added to a 27.5 μL test sample. This mixture was incubated in the dark and at room temperature for 30 min. Gallic and ascorbic acids were used as standard antioxidant agents in the experiments for comparison. Absorbance values were recorded at 450 nm, and the results were calculated from triplicate experiments as in Table 11.

## Supporting Information Available

Preparation of essential oil standards for TLC according to PhEur with modifications; TLC image of *L. latifolia* essential oil and its fractions; TLC image of *R. officinalis* essential oil and its fractions; TLC image of *S. sclarea* essential oil and fractions; TLC image of *M. chamomilla* essential oil and its fractions; and droplet size distribution of essential oil emulsions (PDF)
