# Supplementary material for: Enhanced Bioactivity of Rosemary, Sage, Lavender, and Chamomile Essential Oils by Fractionation, Combination, and Emulsification
Source: ACS Omega. 2023 Mar 14;8(12):10941–53. doi: 10.1021/acsomega.2c07508 (PMC10061596; doi:10.1021/acsomega.2c07508)
Supplement: Supplementary file 1 — ao2c07508_si_001.pdf [file ao2c07508_si_001.pdf]

Supporting Information

Enhanced Bioactivity of Rosemary, Sage, Lavender, and Chamomile Essential Oils by Fractionation, Combination, and Emulsification

Nursenem Karaca[a], Betül Demirci[b], Mohsen Gavahian[c] and Fatih Demirci[b,d*]

[a]*Department of Pharmacognosy, Graduate School of Health Sciences, Anadolu University, Eskisehir, Türkiye.*

[b]*Department of Pharmacognosy, Faculty of Pharmacy, Anadolu University, Eskisehir, Türkiye.*

[c]*Department of Food Science, National Pingtung University of Science and Technology, 1, Shuefu Road, Neipu, Pingtung, 91201, Taiwan.*

[d]*Faculty of Pharmacy, Eastern Mediterranean University, Famagusta, N.Cyprus, Mersin 10, Türkiye*.

***Corresponding Author**

Address: Department of Pharmacognosy, Faculty of Pharmacy, Anadolu University, Eskisehir, Türkiye; Faculty of Pharmacy, Eastern Mediterranean University, Famagusta, N.Cyprus, Mersin 10, Türkiye.

E-mail: fdemirci@anadolu.edu.tr, demircif@gmail.com

Table S1. Preparation of essential oil standards for TLC according to PhEur with modifications

Figure S1. TLC image of *L. latifolia* essential oil and its fractions

Figure S2. TLC image of *R. officinalis* essential oil and its fractions

Figure S3. TLC image of *S. sclarea* essential oil and fractions

Figure S4. TLC image of *M. chamomilla* essential oil and its fractions

Figure S5. Droplet size distribution of essential oil emulsions

TABLE

Table S1. Preparation of essential oil standards for TLC according to PhEur with modifications

***L. latifolia* essential oil**	***R. officinalis* essential oil**	***S. sclarea* essential oil**	***M. chamomilla* essential oil**
10 µL 1,8-cineol<br>10 µL linalool<br>10 µL linalyl acetate<br>1 mL toluene	50 mg borneol<br>50 mg bornyl acetate<br>100 µL 1,8-cineol<br>9.9 mL toluene	60 µL linalool<br>200 µL linalyl acetate<br>60 µL *α*-terpineol<br>7.8 mL toluene	10 mg/mL<br>(-)-bisabolol<br>10 mg/mL farnesene isomers<br>1 mL *n*-hexane

FIGURES

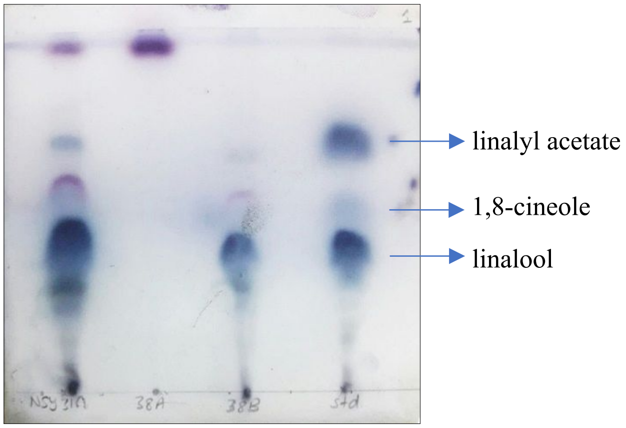

Figure S1. TLC image of *L. latifolia* essential oil and its fractions (from left to right); *L. latifolia* essential oil, *n*-hexane fraction, diethyl ether fraction and standard solution

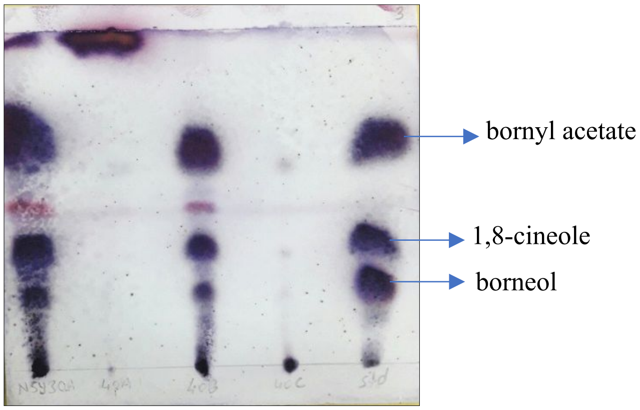

Figure S2. TLC image of *R. officinalis* essential oil and its fractions; *R. officinalis* essential oil, *n*-hexane fraction, diethyl ether fraction, methanol fraction and standard solution (left to right)

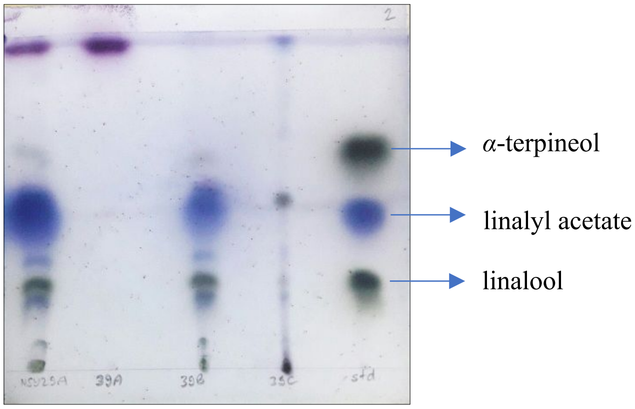

Figure S3. TLC image of *S. sclarea* essential oil and fractions (left to right); *S. sclarea* essential oil, *n*-hexane fraction, diethyl ether fraction, methanol fraction and standard solution.

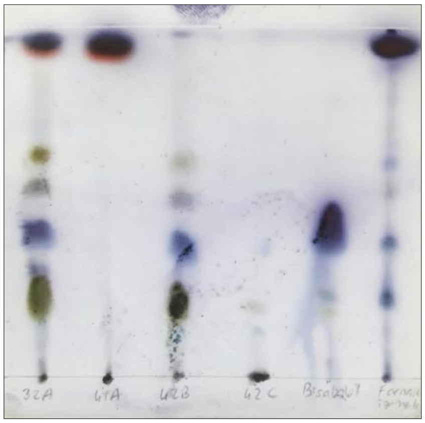

Figure S4. TLC image of *M. chamomilla* essential oil and its fractions (left to right); *M. chamomilla* essential oil, *n*-hexane fraction, diethyl ether fraction, methanol fraction, (-)-bisabolol, farnecene isomers.

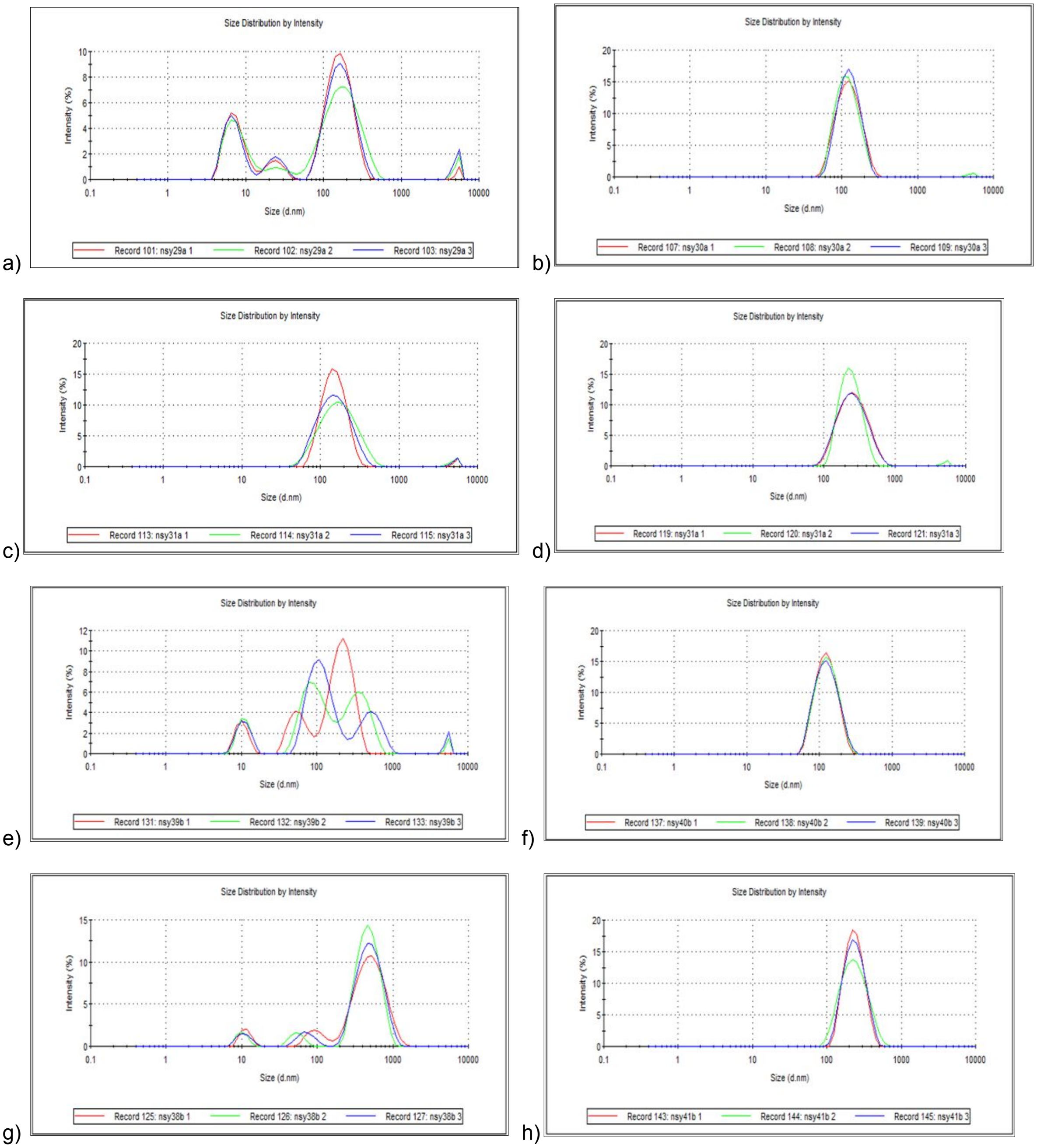

Figure S5. Droplet size distribution of essential oil emulsions

a) *R. officinalis*, b) *S. sclarea*, c) *L. latifolia*, d) *M. chamomilla*, e) *R. officinalis* diethyl ether fraction, f) *S. sclarea* diethyl ether fraction, g) *L. latifolia* diethyl ether fraction, h) *M. chamomilla* diethyl ether fraction
